# Dynamic Hydrogels with Viscoelasticity and Tunable Stiffness for the Regulation of Cell Behavior and Fate

**DOI:** 10.3390/ma16145161

**Published:** 2023-07-21

**Authors:** Yuhang Zhang, Zhuofan Wang, Qingqing Sun, Qian Li, Shaohui Li, Xiaomeng Li

**Affiliations:** 1 School of Mechanics and Safety Engineering, Zhengzhou University, Zhengzhou 450001, Chinaqianli@zzu.edu.cn (Q.L.); 2 National Center for International Joint Research of Micro-Nano Moulding Technology, Zhengzhou University, Zhengzhou 450001, China; 3 School of Materials Science and Engineering, Zhengzhou University, Zhengzhou 450001, China

**Keywords:** dynamic mechanical microenvironment, hydrogel, viscoelasticity, dynamic stiffness

## Abstract

The extracellular matrix (ECM) of natural cells typically exhibits dynamic mechanical properties (viscoelasticity and dynamic stiffness). The viscoelasticity and dynamic stiffness of the ECM play a crucial role in biological processes, such as tissue growth, development, physiology, and disease. Hydrogels with viscoelasticity and dynamic stiffness have recently been used to investigate the regulation of cell behavior and fate. This article first emphasizes the importance of tissue viscoelasticity and dynamic stiffness and provides an overview of characterization techniques at both macro- and microscale. Then, the viscoelastic hydrogels (crosslinked via ion bonding, hydrogen bonding, hydrophobic interactions, and supramolecular interactions) and dynamic stiffness hydrogels (softening, stiffening, and reversible stiffness) with different crosslinking strategies are summarized, along with the significant impact of viscoelasticity and dynamic stiffness on cell spreading, proliferation, migration, and differentiation in two-dimensional (2D) and three-dimensional (3D) cell cultures. Finally, the emerging trends in the development of dynamic mechanical hydrogels are discussed.

## 1. Introduction

Cells live in a complex extracellular microenvironment, also known as the cellular niche. The extracellular microenvironment is composed of the extracellular matrix (ECM), extracellular vesicles (EVs), and growth factors (GFs), which play distinct key roles in determining cellular biological processes [1,2,3,4]. The ECM provides cells with a network structure and biochemical and biophysical cues, thereby regulating various cellular behaviors during life processes [5,6,7,8,9]. Previous studies have indicated that biochemical cues, such as growth factors and bioactive ligands, can regulate cellular activities [10,11,12,13]. Recently, an increasing body of evidence suggests that biophysical cues, particularly the mechanical properties of the ECM, can greatly influence cellular behavior and fate [14,15,16,17,18].

Hydrogels have been widely used to mimic the ECM for cell culture, and their stiffness can impact cell spreading [19,20,21], proliferation [22,23,24,25], migration [26,27,28], and differentiation [29,30,31]. However, native ECM exhibits dynamic mechanical features, such as viscoelasticity [32,33,34,35] and dynamic stiffness [36,37,38,39]. Viscoelasticity refers to the instantaneous elastic response and time-dependent energy dissipation of tissues when subjected to loads. Dynamic stiffness refers to the variation in tissue stiffness during processes such as growth, development, and disease. The mechanical microenvironment of viscoelasticity and dynamic stiffness can better simulate the dynamic mechanical characteristics of native ECM and explore the relationship between dynamic mechanics and cellular behavior and fate.

With the development of hydrogel biomaterials, researchers have developed various types of viscoelastic and dynamic stiffness hydrogels to construct dynamic mechanical microenvironments. Viscoelastic hydrogels encompass physically crosslinked hydrogels (e.g., ion bonding [40,41,42], hydrogen bonding [43,44,45], hydrophobic interactions [46,47,48], and supramolecular interactions [49,50,51]) and dynamically covalently crosslinked hydrogels [52,53,54]. Dynamic stiffness hydrogels mainly include three types: dynamically softening hydrogels [55], dynamically stiffening hydrogels [56], and dynamically reversible stiffness hydrogels [57,58]. Numerous studies have demonstrated that the time-dependent mechanical properties (viscoelasticity and dynamic stiffness) of the ECM can have significant impacts on various essential biological processes [59,60,61,62].

Although there are some reviews summarizing dynamic mechanical hydrogels, most of them mainly focus on viscoelastic hydrogels, and there is a lack of detailed distinction regarding the influence of viscoelasticity on cell behavior in different culture formats (2D and 3D) [63,64,65,66]. In this review, we first emphasize the significance of tissue viscoelasticity and dynamic stiffness. Then, we systematically describe the techniques for characterizing viscoelasticity and stiffness at both macroscopic and microscopic scales. Subsequently, we summarize the current methods for creating viscoelastic and dynamic stiffness hydrogels via physical and chemical crosslinking. We also highlight the regulatory role of the dynamic mechanical microenvironment on cell spreading, proliferation, migration, and differentiation in 2D and 3D cell cultures. Lastly, we provide a future perspective on the mechanical properties of dynamic hydrogels. We aim to provide an all-inclusive overview of viscoelastic and dynamic stiffness hydrogels’ applications in cell behavior and fate. Additionally, we hope this article will inspire researchers to advance the development of novel dynamic mechanical hydrogels and encourage research on cell–matrix interactions.

## 2. Dynamic Mechanical Properties of Tissue

Viscoelasticity and dynamic stiffness are widely present in natural tissues and play crucial roles in various life processes, including development and physiology. In this section, we will introduce the viscoelastic and dynamic stiffness properties of tissues and their significance.

### 2.1. Viscoelasticity of Biological Tissue

Biological tissues have been widely confirmed to be viscoelastic, including the brain [67,68,69], lungs [70], skin [71,72], liver [73,74], muscles [75,76], bones [77], cartilage [78], tendons [79,80], and trachea [81]. The viscoelasticity of biological tissues is mainly determined by the main tissue components, cells and complex ECM. Living cells in biological tissues exhibit viscoelasticity by deforming under load without compromising their integrity [82]. When cells sense forces, the cellular cytoskeleton undergoes dissociation and remodeling, which is the main reason for cell viscoelasticity [83]. The ECM contains fibrous structural proteins, adhesive proteins, and polysaccharides. It is a major component in tissues and forms a complex three-dimensional network. The dissociation and reorganization of the non-covalent weakly bonded crosslinked collagen fibril network and the release of polymer entanglements facilitate the sustained dissipation of energy, which significantly increases tissue viscosity [32]. Finally, tissue viscoelasticity is also attributed to the exchange and movement of fluids within the porous microstructure [84], such as the heart, kidneys, and liver [85,86]. The viscoelasticity derived from fluid movement is closely related to the pore size and porosity of living tissues. Because the structure and composition of each tissue are different, the relative importance of these dissipative mechanisms may vary in different tissues.

Natural tissues exhibit viscoelastic behavior and play a crucial role in maintaining physiological functions. For instance, the viscoelastic properties of tendons enable them to store and release energy, preventing excessive stretching and damage during movement processes [87,88]. The viscoelastic properties of cartilage allow it to effectively absorb impact and disperse pressure. When joints are subjected to impact or load, cartilage can deform and rebound, reducing pressure on the joints and bones, thereby protecting the joint surfaces from damage [89]. The viscoelasticity of the brain contributes to supporting and protecting brain tissue, stabilizing the position and connections of neurons and maintaining normal neuronal activity and neural signal transmission [90]. Changes in the viscoelasticity of the brain may be associated with the development and progression of various neurological disorders and neurodegenerative diseases. For instance, conditions such as Alzheimer’s disease, Parkinson’s disease, concussion, and brain injury can potentially lead to alterations in the viscoelastic properties of the brain [91,92,93]. Both Alzheimer’s and Parkinson’s diseases result in a decrease in local brain viscosity compared to healthy brains. By studying changes in brain viscoelasticity, the pathophysiological process of these diseases can be better understood, providing new methods and strategies for early diagnosis and treatment [94,95]. Similarly, the viscoelastic properties of the liver can also reflect changes in its pathological state [96]. The viscoelastic parameters of liver tissue are positively correlated with the percentage of fibrosis, with the viscoelastic parameters increasing as the degree of liver fibrosis increases [97]. Measuring the viscoelasticity of the liver can provide information about the progression of liver diseases and the effectiveness of treatments [98]. Therefore, gaining a deep understanding of the viscoelastic mechanical properties of tissues and studying their impact on physiological functions, tissue homeostasis, and disease progression can provide novel strategies for the diagnosis and treatment of clinical diseases in the future.

### 2.2. Dynamic Stiffness of Biological Tissue

Stiffness is defined as the degree to which a material resists deformation in response to an applied force. In biology, stiffness has been used to represent the mechanical properties of biological matrices. Our tissues are composed of a variety of different ECM molecules; each tissue/organ has a specific stiffness to meet physiological needs [99]. In addition, the stiffness of tissues will also change during the processes of development, regeneration, fibrosis, and movement. During the first six months of a rabbit’s life, the modulus of elasticity of rabbit bones increases by 55–65% [100]. The stiffness of the bone is determined by its mineral content. With an increase in mineralization, the Young’s modulus of the bone will also increase. The collagen in the bone determines its toughness, and a decrease in collagen content makes the bone more brittle without altering its stiffness [101]. The reduction in loosely bound water at the interface between collagen and mineral components decreases the bone’s ability to dissipate energy [102]. During the embryonic development of the mouse brain, the Young’s moduli of the ventricular zone (VZ) and subventricular zone (SVZ) increase by 128.78% and 219.07%, respectively [103]. Changes in stiffness may be related to the transition from neurogenesis to gliogenesis later in embryonic brain development. Therefore, changes in tissue stiffness during development are related to tissue maturation. During aging and disease, there are corresponding changes in tissue stiffness. Five days after tibialis anterior (TA) injury, the modulus of elasticity dropped by half (5 kPa). As regeneration progressed, muscle stiffness increased to double (19.3 kPa) 14 days after injury. There was a continued increase in stiffness (22.1 kPa) for at least 28 days after injury [104]. In age-matched individuals, the stiffness of the trabecular meshwork (TM) is significantly greater in glaucomatous patients than in normal individuals [105]. SC endothelial cells from glaucoma patients are enriched in genes involved in ECM remodeling and cell adhesion, which may contribute to increased cell stiffness. Local stiffness increased by more than 5-fold after pulmonary fibrosis compared with normal lung [106]. Hepatic fibrosis leads to an increase in spleen stiffness. This is primarily due to passive congestion, sinus dilation, diffused αSMA expression of sinusoidal mesenchymal cells, and deposition of collagen fibers on the perisinusoidal wall in the spleen tissue. [107]. During exercise, moderate to rapid walking (3.6 mph) decreased plantar tissue stiffness compared with slow walking (1.8 mph), which may decrease the risk of foot ulcers [108]. Proper exercise intensity provides the proper amount of physical stress to maintain tissue health. Tissue damage can occur when the intensity of exercise is too low or too high. Therefore, by studying the dynamic stiffness of tissues, we can gain a deep understanding of the mechanical response and deformation behavior of tissues, as well as the impact of these properties on cellular behavior and fate. This is of great significance for a comprehensive understanding of physiological and pathological processes and for providing new strategies for the diagnosis and treatment of clinical diseases.

## 3. Multiscale Characterization of Dynamic Mechanics

The dynamic mechanical microenvironment of natural tissues plays a crucial role in maintaining tissue function and regulating cellular behavior. This microenvironment primarily includes the viscoelasticity and dynamic stiffness of the tissue. Hydrogels have been widely used to simulate the dynamic mechanical properties of tissues and study their effects on cellular behavior. With the rapid advancement of technology, various characterization techniques have been developed to assess the dynamic mechanical properties of materials and tissues, including macroscopic characterization and microscopic characterization.

### 3.1. Macroscopic Characterization

**Figure 1:**
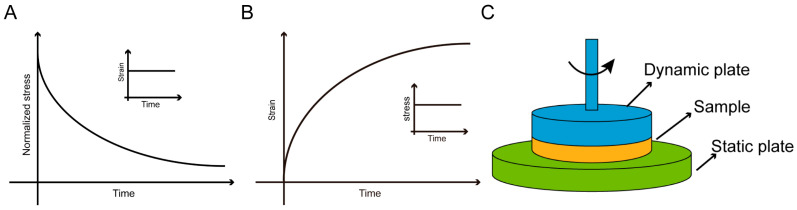
Macroscopic characterization of mechanical properties. (**A**) Stress relaxation test. (**B**) Creep test. (**C**) Frequency-dependent rheology test.

Static mechanical testing and dynamic mechanical testing are common methods for characterizing the macroscopic mechanical properties of materials. In static mechanics testing, compression experiments can be used to characterize the stiffness and viscoelasticity of materials. The sample is placed between two parallel plates for compression experiments, and the Young’s modulus (corresponding to the elastic modulus) is obtained by linearly fitting the stress–strain curve, which represents the stiffness of the material. For instance, this method allows for the testing of the stiffness of sodium alginate hydrogels with varying crosslinking strength [109]. Stress relaxation is the decrease in stress over time when a constant strain is applied to a material (Figure 1A). Stress relaxation can characterize the viscoelastic properties of materials, such as the rapid stress relaxation exhibited by human hematomas [41]. Creep is defined as the increase in strain over time when a constant stress is applied to a material (Figure 1B). The creep behavior of PAM-co-PDAAM polymer hydrogels containing yeast is influenced by pH. The acylhydrazone bonds are highly stable under neutral and alkaline pH conditions (typically above 7), while they exhibit rapid acylhydrazone exchange under acidic pH conditions (typically below 6) [110]. In both of these static methods, time is typically considered as a parameter for stress relaxation or creep rate. τ_1/2_ is defined as the time required for the initial stress value to decrease by half. τ_3/2_ is defined as the time required to reach 150% of the initial strain. Therefore, by comparing the τ_1/2_ and τ_3/2_ parameters of different materials, the viscoelasticity of the materials can be quantitatively analyzed.

Frequency-dependent rheological tests are the most widely used dynamic mechanical testing method (Figure 1C). Applying sinusoidal strain at different frequencies will result in a corresponding stress response. There is a phase shift between the applied sinusoidal strain and the stress response. The in-phase response reflects the energy stored by the material under cyclic loading, representing the material’s elastic response to strain, known as the elastic modulus or storage modulus (G′). On the other hand, the out-of-phase response represents the material’s ability to dissipate energy under cyclic loading, reflecting the material’s dissipative response to strain, known as the viscous or loss modulus (G″). The storage modulus and loss modulus are two important parameters used to describe the dynamic mechanical properties of materials at different frequencies [111]. Typically, viscoelastic materials exhibit frequency-dependent behavior, while elastic materials maintain relatively constant mechanical properties [112]. For example, viscoelastic GelNB-BA hydrogels exhibit an increase in storage modulus and loss modulus with increasing frequency [113]. In addition, cyclic loading tests can also be used to characterize the viscoelasticity of materials. The presence of a hysteresis loop is due to the viscoelasticity of the material and its associated energy dissipation. The area enclosed by the hysteresis loop reflects the viscosity of the viscoelastic hydrogel. For instance, significant energy dissipation capacity was observed in glycerol-crosslinked PVA hydrogels (GPG) during continuous cyclic compression over 100 cycles [114].

### 3.2. Microscopic Characterization

Macroscopic mechanical characterization techniques can only collect the overall mechanical information of materials. However, there are situations when we need to acquire mechanical information at the local scale. The characterization of mechanical properties at the microscale has become increasingly mature as material characterization techniques have advanced rapidly. This mainly includes indentation-based techniques, micro-rheology, and elastic imaging. These techniques allow for mechanical testing and analysis of materials at the micrometer scale, providing mechanical information such as local stiffness, elastic modulus, and viscoelasticity.

**Figure 2:**
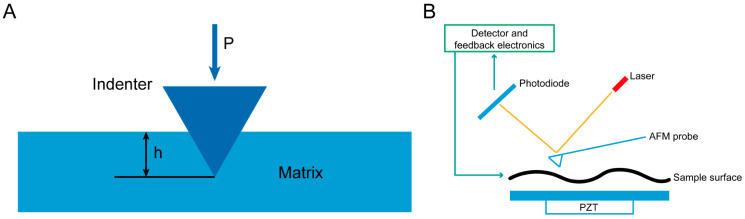
Microscale mechanical characterization of indentation. (**A**) Depth-sensing nanoindentation. (**B**) AFM-based indentation.

Indentation-based technologies mainly include depth-sensing nanoindentation and atomic force microscopy (AFM)-based indentation. For depth-sensing nanoindentation, a calibrated force is applied through the probe tip to directly measure the displacement of the probe in the plane perpendicular to the sample. During the loading process, the initial response on the sample surface is elastic deformation. As the load increases, plastic deformation gradually appears and grows. During the unloading process, the main mechanism is the recovery of elastic deformation, while the residual plastic deformation results in the formation of an indentation on the sample surface (Figure 2A) [115]. This method has the characteristics of high resolution, non-destructiveness, and a wide range of applicability. By calculating the slope of the force–displacement curve, the elastic modulus of the material can be determined, which represents its stiffness [116]. The stress–time curve is used to characterize the stress relaxation rate of hydrogel [117], while the displacement–time curve is used to characterize the creep behavior of human tooth enamel [118]. Nanomechanical indentation also enables measurements like dynamic mechanical analysis (DMA) by using mechanical oscillations. Through frequency scanning, it allows the quantification of the viscoelastic properties (G′ and G″) of hydrogels [119]. The results indicate that the loss modulus of oligo(ethylene glycol) (OEG) hydrogels exhibits frequency dependence.

**Figure 3:**
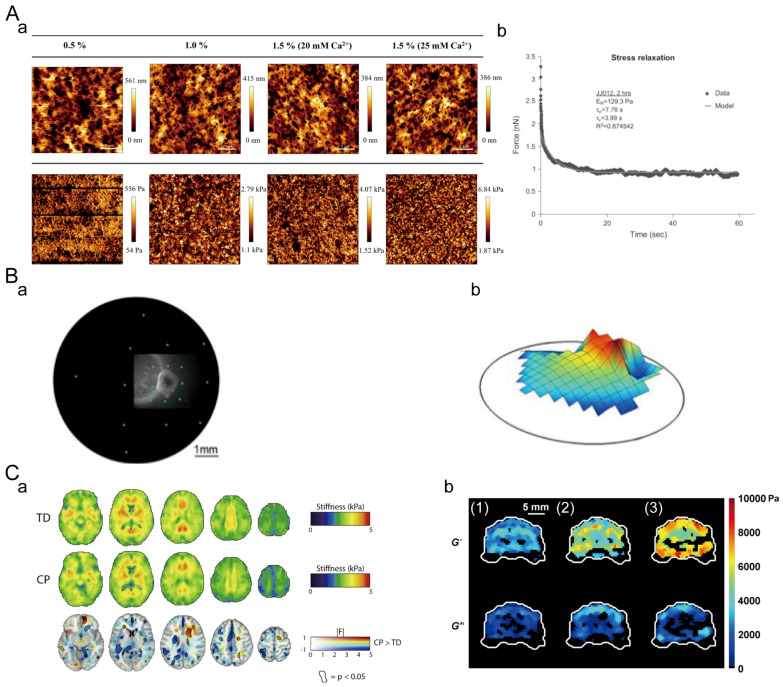
Microscopic characterization of mechanical properties. (**A**) AFM-based indentation test. (**a**) Local stiffness of alginate hydrogels. Adapted with permission from [120]. Copyright 2022, Elsevier. (**b**) Stress relaxation of chondrosarcoma cells and stress relaxation response with thin-layer viscoelastic model. Adapted with permission from [121]. Copyright 2007, Elsevier. (**B**) Particle-based micro-rheology. (**a**) PANC-1 3D particles in collagen ECM. (**b**) Spatial stiffness map of the ECM. Adapted with permission from [123]. Copyright 2014, JoVE. (**C**) Elastography. (**a**) Brain stiffness of normal children (TD) and children with cerebral palsy (CP). Adapted with permission from [69]. Copyright 2019, Elsevier. (**b**) Viscoelasticity of the ferret brain for 400 Hz (**1**), 600 Hz (**2**) and 800 Hz (**3**). Adapted with permission from [125]. Copyright 2013, Elsevier.

For AFM-based indentation, a microcantilever, highly sensitive to extremely weak forces, is fixed at one end while having a small needle tip at the other end. The needle tip gently contacts the surface of the sample. The microcantilever with the needle tip undergoes slight vertical oscillation in the direction perpendicular to the sample surface. There exists an extremely weak repulsive force between the atoms at the tip of the needle and the atoms on the sample surface. By controlling this force to remain constant during scanning, an equipotential surface corresponding to the interatomic forces is generated. Optical detection or tunneling current detection can be used to measure the positional changes corresponding to each point of the scan. The signal is amplified and converted to obtain a three-dimensional topographical image of the sample surface at the atomic level (Figure 2B). The AFM-based nanoindentation mechanical testing has advantages such as high resolution, no need for large samples, and multifunctionality. The force curve obtained from AFM can be used to calculate the local Young’s modulus of sodium alginate hydrogel (Figure 3A(a)) [120]. This technique can also be employed to characterize the microscale viscoelasticity. For example, stress relaxation tests of chondrosarcoma cells at the microscale were conducted using AFM. The results revealed that chondrosarcoma cells exhibited viscoelastic properties (Figure 3A(b)) [121].

Particle-based micro-rheology involves dispersing micrometer-sized particles in the material of interest and tracking the displacements of these particles. The restricted motion of the tracer particles is reflected in the plateau value of the mean square displacement. This plateau value corresponds to the maximum displacement and depends on the local environment’s stiffness. Using the generalized Stokes–Einstein equation, the measured mean square displacement can be calculated as the frequency-dependent storage modulus G′(ω) and loss modulus G″(ω). This approach has several advantages, including a quick data-collecting time and high spatiotemporal resolution. This particle-based micro-rheology includes passive and active micro-rheology techniques [122]. The active method refers to the use of internal stress generated by the free movement of particles in the material to be tested, and the passive method refers to the application of external stress (rotating magnetic field or mechanical stress device) to the test material. Dustin P. Jones et al. encapsulated multiple detectable PANC-1 3D spheroids in collagen ECM (Figure 3B) [123]. By combining the coordinate data with the calculated G′(ω) for each position, spatial ECM stiffness maps can be generated. Region two exhibits a liquid-like viscoelastic response, as is evident by the viscous scaling of the G″(ω) plot and the absence of the fit line for G′(ω). Because consistently dispersing probe particles throughout dense tissue samples without harming the specimen is difficult, this approach is limited to use in very soft tissues.

Elastic imaging enables non-invasive measurements of tissue mechanical properties [124]. In elastic imaging experiments, shear waves are generated using ultrasound or magnetic resonance to introduce low-frequency vibrations into the sample. The deformation of the tissue under the vibrational load is then measured, and mathematical algorithms are used to convert the deformation into a tissue stiffness image. Elastic imaging has the advantages of high spatial resolution, non-destructiveness, and real-time imaging. Non-invasive measurement of brain stiffness using magnetic resonance imaging revealed significantly lower stiffness in the brains of children with cerebral palsy compared to normal brains (Figure 3C(a)) [69]. The deformation response of the samples can also be used to calculate the viscoelastic parameters of the samples using mathematical models. For example, testing the ferret brain showed that both the storage modulus and loss modulus of the brain increased with increasing frequency (Figure 3C(b)) [125]. However, this technique is only applicable for scanning large-scale tissues and cannot provide reliable information at the cellular level.

## 4. Construction of Hydrogels with Dynamic Mechanical Properties

Hydrogel is a highly hydrated material with a three-dimensional network structure and excellent biocompatibility and has been widely used in tissue engineering. Currently, research in the field of biomaterial mechanics is gradually shifting from static mechanics to dynamic mechanics. Hydrogels can achieve the dynamic mechanical properties of natural ECM through special crosslinking methods, which can be used to study the regulation of ECM dynamic mechanics on cell behavior and fate.

In recent years, dynamic hydrogels based on various crosslinking strategies have been created. Viscoelastic hydrogel crosslinking methods include physical crosslinking (such as ionic bonds, hydrogen bonds, hydrophobic interactions, and host–guest reactions) and dynamic covalent crosslinking. Dynamic stiffness hydrogels mainly include hydrogels with dynamic softening, dynamic stiffening, and dynamic reversible stiffness.

### 4.1. Crosslinking Strategies for Viscoelastic Hydrogels

#### 4.1.1. Ionically Crosslinked Hydrogels

**Figure 4:**
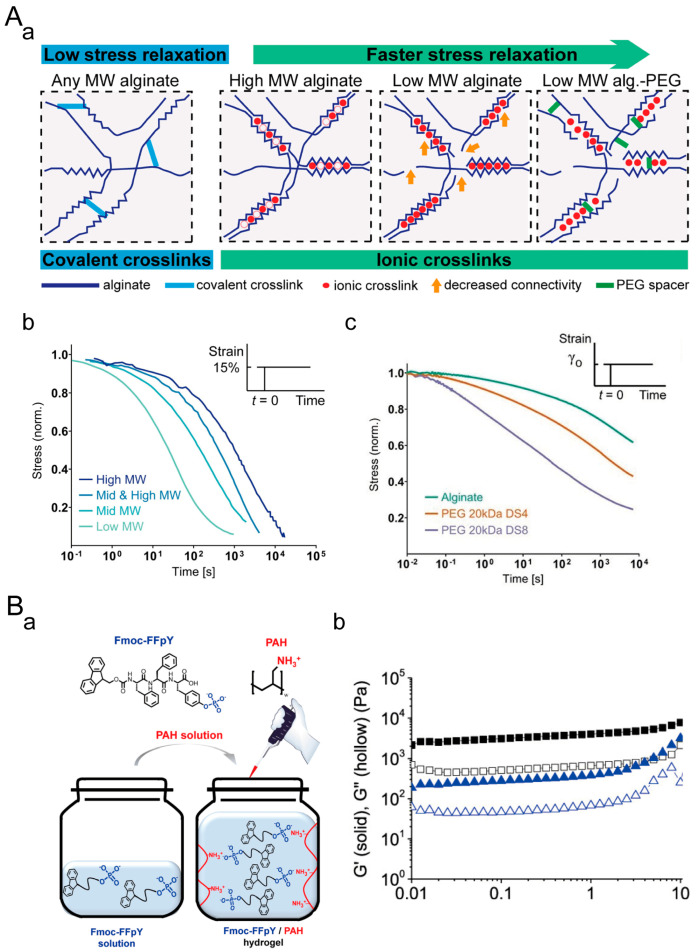
Ionically crosslinked viscoelastic hydrogels. (**A**) Sodium alginate hydrogel crosslinked by calcium ions. (**a**) The stress relaxation of sodium alginate hydrogels can be regulated by altering the crosslinking type and molecular weight of sodium alginate. (**b**) Stress relaxation of sodium alginate hydrogels crosslinked with calcium ions of different molecular weights. (**c**) Stress relaxation of sodium alginate hydrogels grafted with varying amounts of PEG chains. Adapted with permission from [126], Copyright 2021, Wiley-VCH GmbH. (**B**) PAH/Fmoc-FFpY supramolecular hydrogels crosslinked by electrostatic interaction. (**a**) The crosslinking principle of PAH/Fmoc-FFpY hydrogels. (**b**) Rheological testing of PAH/Fmoc-FFpY (■) and Fmoc-FFY (▲) hydrogels. Solid symbols represent G′ and hollow symbols represent G″. Adapted with permission from [127]. Copyright 2020, American Chemical Society.

Ionically crosslinked hydrogels have advantages such as simplicity, tunability, and biocompatibility. However, they also have disadvantages, including ion sensitivity, limitations in mechanical properties, and challenges in controllability. The most common ion-crosslinked hydrogel is calcium-ion-crosslinked sodium alginate hydrogel, and CaCl_2_, CaSO_4_, or CaCO_3_ can all crosslink sodium alginate. Among them, when CaCl_2_ crosslinks bulk gels, the crosslinked network will be inhomogeneous. Both CaCO_3_ and CaSO_4_ will be crosslinked to form a uniform hydrogel, but when CaCO_3_ crosslinks sodium alginate, air bubbles will be generated in the hydrogel. Frank Charbonier et al. developed calcium-ion-crosslinked alginate hydrogels with different viscoelastic properties (Figure 4A) [126]. Compared to covalently crosslinked elastic sodium alginate hydrogels, calcium-ion-crosslinked sodium alginate hydrogels exhibit rapid stress relaxation, and as the molecular weight of alginate decreases, the chain mobility increases, resulting in faster stress relaxation rates. The presence of polyethylene glycol (PEG) chains can impede the calcium ion crosslinking of alginate hydrogels. By varying the total amount of PEG, the stress relaxation of calcium-ion-crosslinked sodium alginate hydrogels can be further controlled. In addition to traditional ionically crosslinked hydrogels, polyelectrolyte hydrogels also exhibit viscoelastic behavior. Miryam Criado-Gonzalez et al. prepared PAH/Fmoc-FFpY supramolecular hydrogels through supramolecular interactions between Fmoc-FFpY peptides and electrostatic interactions between the peptides and oppositely charged polyelectrolyte chains (Figure 4B) [127]. The self-assembly of the hydrogels was driven by electrostatic interactions. These hydrogels have higher mechanical properties compared to Fmoc-FFY hydrogels produced through peptide dephosphorylation.

#### 4.1.2. Hydrogen-Bonded Crosslinked Hydrogels

**Figure 5:**
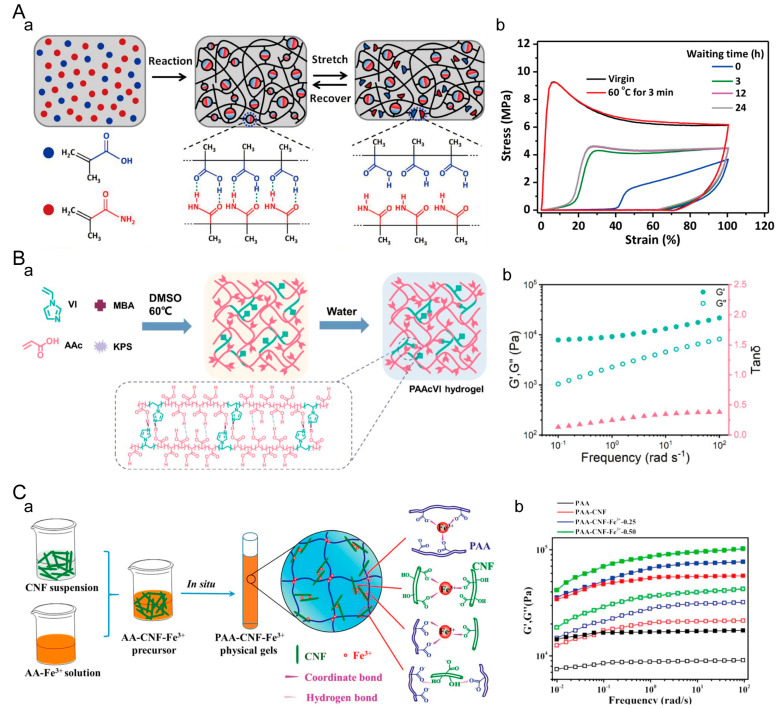
Hydrogen-bonded crosslinked hydrogels. (**A**) Hydrogen-bonded P(MAAm-co-MAAc) dynamic hydrogel. (**a**) Crosslinking principle of P(MAAm-co-MAAc) hydrogel. (**b**) Cycling loading test of P(MAAm-co-MAAc) hydrogel. Adapted with permission from [129]. Copyright 2019, American Chemical Society. (**B**) PAAcVI hydrogel with synergistic hydrogen bonding and electrostatic interactions. (**a**) Crosslinking principle of PAAcVI hydrogel. (**b**) Frequency-dependent rheological test of PAAcVI hydrogel. Adapted with permission from [130]. Copyright 2022, American Chemical Society. (**C**) PAA-CNF-Fe^3+^ hydrogel with synergistic hydrogen and coordination bonding. (**a**) Crosslinking principle of PAA-CNF-Fe^3+^ hydrogel. (**b**) Frequency-dependent rheological test of the hydrogel. Solid symbols represent G′ and hollow symbols represent G″. Adapted with permission from [131]. Copyright 2017, American Chemical Society.

In the dynamic bonding mechanism, hydrogen bonding is a very common interaction. Hydrogen-bonded crosslinked hydrogels have the advantages of controllability and reversibility. However, they also have weak mechanical properties and high sensitivity to environmental factors such as temperature and pH. Since individual hydrogen bonds in hydrogel systems are typically weak, it is generally achieved by creating multiple multivalent hydrogen bonds or synergistically combining them with other types of chemical interactions to form a strong network [128]. Yan Jie Wang et al. prepared a dynamic hydrogel, P(MAAm-co-MAAc), by utilizing strong hydrogen bonding reactions between MAAm and MAAc (Figure 5A) [129]. The hydrogel exhibited significant hysteresis in the first loading–unloading curve with a maximum strain of 100%, indicating the dissipation of a considerable amount of energy during the loading process. Xiaodong Wang et al. prepared poly(acrylic acid-co-1-vinylimidazole) (PAAcVI) hydrogels by using acrylic acid (AAc) and 1-vinylimidazole (VI) as monomers in dimethyl sulfoxide (DMSO) [130]. Subsequently, solvent exchange was carried out with water. The PAAcVI hydrogels were prepared due to the strong hydrogen bonding and electrostatic interactions between the carboxylic acid and imidazole groups in the hydrogel (Figure 5B). In frequency sweep tests of PAAcVI hydrogels at 25 °C, the storage modulus (G′) and loss modulus (G″) exhibit their minimum values near a frequency of 1 rad/s and increase with increasing frequency. This behavior is attributed to the synergistic effect of hydrogen bonding and strong electrostatic interactions. Changyou Shao et al. induced the formation of a dynamic crosslinked network by using cellulose nanofibers (CNFs) to form hydrogen bonds with poly(acrylic acid) (PAA) chains, followed by secondary crosslinking through dual ion coordination bonds formed between Fe^3+^ ions and the carboxyl groups of both PAA and carboxylated CNFs (Figure 5C) [131]. The coordination bond is a strong bond that maintains the primary structure, while the hydrogen bond is a weak bond that forms a sacrificial network. Both bonds synergistically contribute to the formation of a dynamic crosslinked network. Hydrogen bonding between the CNF interface and PAA matrix leads to an increase in the storage modulus (G′) and loss modulus (G″) of the PAA-CNF hydrogel with increasing frequency. As the concentration of Fe^3+^ increases, the synergistic effect of coordination bonds significantly enhances the dynamic modulus of the PAA-CNF-Fe^3+^ hydrogel.

#### 4.1.3. Hydrophobic-Interaction-Crosslinked Hydrogels

**Figure 6:**
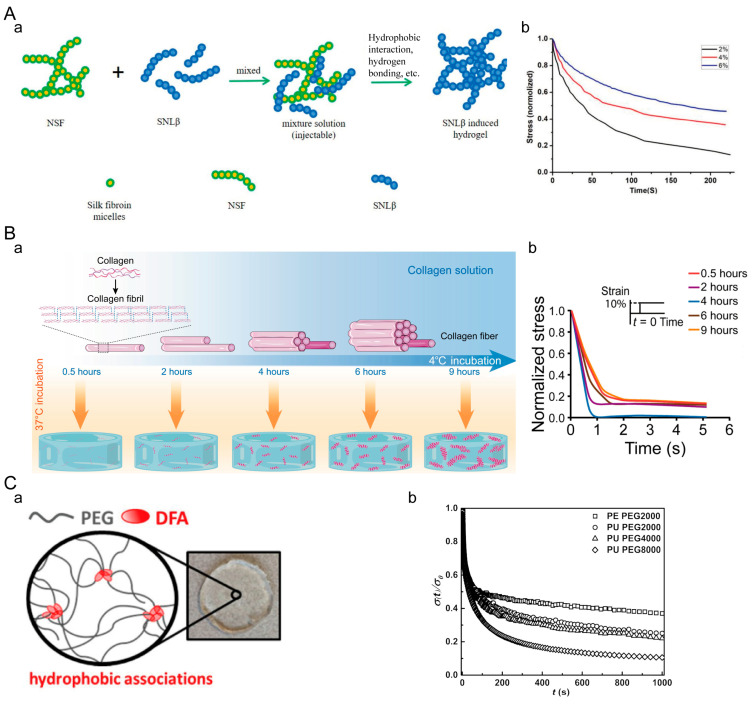
Hydrophobic-interaction-crosslinked hydrogel. (**A**) SNLβ hydrogel. (**a**) Crosslinking principle of SNLβ hydrogel. (**b**) Stress relaxation of SNLβ hydrogel with different silk fibroin concentrations. Adapted with permission from [135]. Copyright 2021, American Chemical Society. (**B**) Collagen hydrogel. (**a**) Crosslinking mechanism of collagen hydrogel. (**b**) Stress relaxation of collagen hydrogel. Adapted with permission from [136]. Copyright 2023, Danyang Huang. (**C**) Tough supramolecular hydrogel based on strong hydrophobic interactions. (**a**) Crosslinking principle of tough supramolecular hydrogel. (**b**) Stress relaxation of hydrogel. Adapted with permission from [137]. Copyright 2017, American Chemical Society.

Hydrogels crosslinked through hydrophobic interactions exhibit reversibility, responsiveness, excellent mechanical properties, and biocompatibility [132,133,134]. By manipulating hydrophobic interactions, we can effectively regulate the mechanical properties of hydrogels, such as viscoelasticity. Therefore, hydrogels crosslinked by hydrophobic interactions are well suited for mimicking the mechanical characteristics of the extracellular microenvironment. Danyu Yao et al. prepared a viscoelastic β-structured nanofiber (SNLβ) hydrogel by mixing a solution of SNLβ with a concentrated solution of nanofiber-containing silk fibroin (NSF) (Figure 6A) [135]. The hydrogel formation was facilitated by a combination of hydrophobic interactions and hydrogen bonding. The dehydration of the silk fibroin led to its gelation, and the addition of SNLβ may have acted as a trigger for gelation. With an increase in the concentration of silk fibroin protein, the stress relaxation rate of the hydrogel significantly accelerates. Danyang Huang et al. achieved a higher degree of self-assembly in collagen fiber networks by prolonging the low-temperature incubation time of a collagen solution (Figure 6B) [136]. At physiological temperature, collagen protein molecules self-assemble into collagen fiber hydrogels through the combination of electrostatic and hydrophobic interactions. Collagen protein hydrogels exhibit viscoelastic behavior as these weak interactions unravel under stress, allowing fiber sliding. As the low-temperature incubation time of collagen protein increases, more weak interactions are established within the collagen fiber network, resulting in an initial acceleration followed by a slowdown of the stress relaxation rate of the collagen protein hydrogel. Marko Mihajlovic et al. reported a simple and fast method for preparing hydrophobic crosslinked supramolecular dynamic hydrogels (Figure 6C) [137]. Due to the strong hydrophobic aggregation between polymer blocks and DFA units, the hydrogel exhibits high mechanical strength, toughness, stability, and viscoelasticity. With an increase in PEG molecular weight, the stress relaxation rate of the hydrogel accelerates. The rapid initial decrease in stress may be related to changes in chain conformation within the network. The subsequent stress gradually decreases, which may be attributed to the escape of DFA units from micelles and subsequent structural reorganization.

#### 4.1.4. Supramolecular Hydrogels

**Figure 7:**
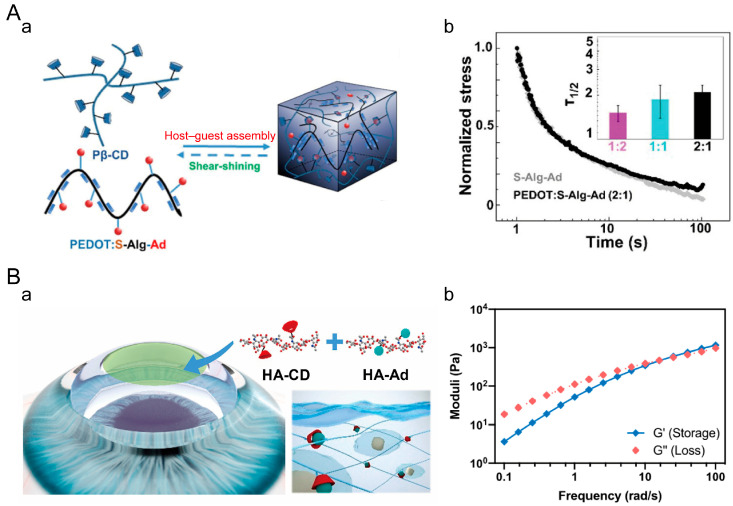
Supramolecular hydrogel. (**A**) Host–guest crosslinked conductive sodium alginate hydrogel. (**a**) Hydrogel crosslinking principle. (**b**) Stress relaxation time of various PEDOT:S-Alg-Ad hydrogels. Adapted with permission from [140]. Copyright 2019, American Chemical Society. (**B**) Host–guest crosslinked s-HA hydrogel. (**a**) Crosslinking principle of the hydrogel promoting corneal wound healing. (**b**) Variation in storage modulus and loss modulus of the hydrogel with frequency. Adapted with permission from [141]. Copyright 2019, Elsevier.

Physically crosslinked viscoelastic hydrogels can also be formed based on the supramolecular recognition of host–guest interactions. The host molecules possess cavity structures, while the guest molecules can be inserted into these cavities. Through this dynamic and reversible host–guest interaction, the host and guest molecules can crosslink with each other, forming a dynamic crosslinked three-dimensional network structure [138]. By utilizing the similar equilibrium binding constants (K_eq_) and kinetic binding constants (k_off_, k_on_) of the host–guest interactions, the viscoelastic properties of the host–guest crosslinked hydrogels can be precisely tuned [139]. Host–guest crosslinked hydrogels have the advantages of tunability, reversibility, and controllable crosslinking sites. However, the synthesis process for these hydrogels is complex and it involves higher costs and resource consumption. Yong Xu et al. designed a conductive sodium alginate hydrogel formed through host–guest interactions between cyclodextrin and adamantane (Figure 7A) [140]. The highly dynamic host–guest interactions facilitated the rearrangement of PEDOT nanoparticles within the network. Hydrogels crosslinked with poly-β-cyclodextrin (Pβ-CD) and different-sized PEDOT:S-Alg-Ad nanoparticles exhibited rapid stress relaxation, indicating that the dynamic network governed by host–guest interactions determined the hydrogel’s dynamic mechanics. Gabriella Maria Fernandes-Cunha et al. designed a supramolecular HA (s-HA) hydrogel formed by host–guest interactions between cyclodextrin (CD) and adamantane (Ad), which possessed the ability to heal corneal wounds (Figure 7B) [141]. The host–guest crosslinked dynamic network exhibited frequency-dependent G′ and G″ and exhibited viscoelastic characteristics.

#### 4.1.5. Dynamic Covalent Hydrogels

**Figure 8:**
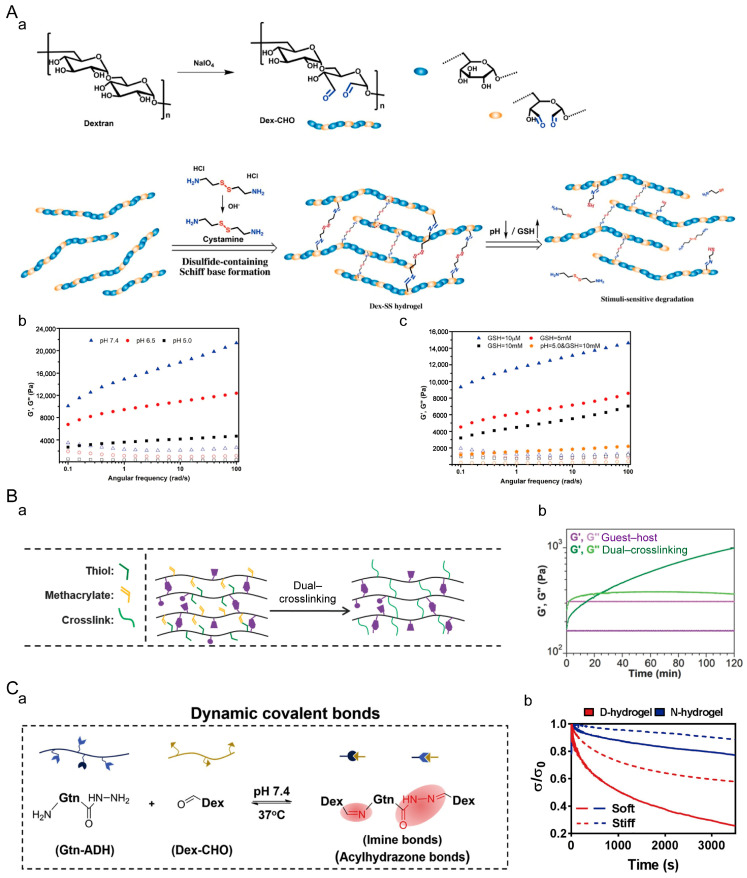
Dynamically covalently crosslinked hydrogels. (**A**) Dextran dynamic hydrogel (Dex-SS). (**a**) Crosslinking mechanism of Dex-SS hydrogel. (**b**) Rheological properties of Dex-SS hydrogel at different pH. (**c**) Rheological properties of Dex-SS hydrogels in different concentrations of GSH. Solid symbols represent G′ and hollow symbols represent G″. Adapted with permission from [157]. Copyright 2021, Elsevier. (**B**) Double-crosslinked hydrogel. (**a**) Crosslinking mechanism of double-crosslinked hydrogels. (**b**) Rheological properties of the hydrogel. Adapted with permission from [158]. Copyright 2014, Wiley-VCH GmbH. (**C**) Dynamic hydrogels crosslinked by imide and acylhydrazone linkages. (**a**) Crosslinking mechanism of dynamic hydrogels. (**b**) Stress relaxation of dynamic hydrogels. Adapted with permission from [159]. Copyright 2020, Elsevier.

Hydrogel containing dynamic covalent chemical bonds, such as thiol-ester [142,143], allyl sulfide [144,145], disulfide [146,147], oxime [54,148], borate ester [149,150], hydrazone [151,152], Schiff base [153,154], and Diels-Alder [155,156], can form covalent adaptable networks, exhibiting viscoelasticity. Dynamic covalent crosslinked hydrogels possess characteristics such as self-healing, responsiveness, injectability, and mechanical strength recoverability. Hongying Su et al. synthesized dextran-based dynamic hydrogels (Dex-SS) containing Schiff base and disulfide bonds by reacting aldehyde-functionalized polyaldehyde dextran (Dex-CHO) with semiamine (Figure 8A) [157]. The viscoelasticity of the hydrogels could be tuned to different degrees by employing acid-catalyzed Schiff base hydrolysis and the sensitive cleavage of disulfide bonds using a reducing agent, glutathione (GSH). Christopher B. Rodell et al. developed a universally applicable dual-crosslinking (DC) approach (Figure 8B) [158]. The first layer of the dynamic network was formed through host–guest crosslinking, followed by the situ Michael addition reaction of MA to form the second layer of the dynamic crosslinked network. The reactivity of the Michael acceptor and the catalytic conditions could be controlled. Compared to the single network formed by the host–guest reaction, the dual network exhibited a higher storage modulus and loss modulus. Zhao Wei et al. utilized the aldehyde groups on dialdehyde-modified dextran (Dex-CHO) and the amino groups on adipic dihydrazide-modified gelatin (Gtn-ADH) to form imine and acyl hydrazone bonds, thereby preparing a dynamic and viscoelastic hydrogel (Figure 8C) [159]. Compared to static hydrogels, this dynamic hydrogel exhibited rapid stress relaxation.

### 4.2. Crosslinking Strategies for Hydrogels with Dynamic Stiffness

The stiffness changes observed in biological tissues during processes such as development, physiology, and disease can be simulated using hydrogels with dynamic stiffness, providing an increasing theoretical foundation for current research in tissue with dynamic mechanics.

#### 4.2.1. Dynamic Softening Hydrogel

**Figure 9:**
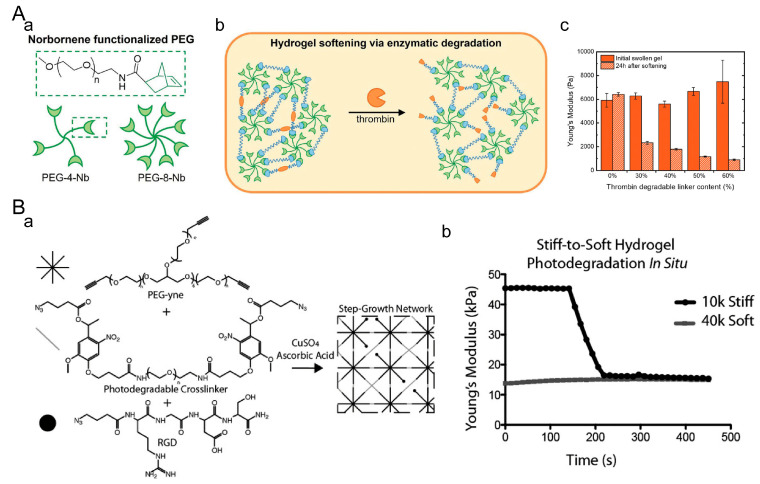
Dynamic softening hydrogels. (**A**) PEG-peptide hydrogel. (**a**) Modification of PEG. (**b**) Softening principle of PEG-peptide hydrogel. (**c**) Influence of thrombin concentration on hydrogel softening. Adapted with permission from [160]. Copyright 2022, Wiley-VCH GmbH. (**B**) Photodegradable hydrogel. (**a**) Principle of photodegradation in the hydrogel. (**b**) Softening of the hydrogel due to photodegradation. Adapted with permission from [161]. Copyright 2014, Wiley-VCH GmbH.

In the study of dynamic mechanical microenvironments, dynamic softening hydrogels can dynamically reduce the stiffness of the hydrogel by degrading the polymer, hydrolyzing the crosslinking chemical bonds, or undergoing photodegradation, thereby reducing the crosslinking strength of the network. Katherine L Wiley et al. developed a tunable polyethylene glycol-peptide hydrogel that can undergo softening (Figure 9A) [160]. The softening capability was achieved by incorporating a dual degradable peptide sequence (MMP + Thb junction) into the hydrogel. By incorporating MMP + Thb junction peptides at different percentages, the hydrogel exhibited the same initial Young’s modulus, while the degree of softening varied depending on the percentage of clotting enzyme-degradable peptides incorporated. Chelsea M. Magin et al. developed a clickable and photodegradable hydrogel system (Figure 9B) [161], which achieved photodegradability by incorporating photo-unstable nitrobenzyl ether moieties into PEG-azide. The photodegradation kinetics are determined by the quantum yield of the photo-unstable nitrobenzyl ether moieties, irradiation intensity, and wavelength of light.

#### 4.2.2. Dynamic Stiffening Hydrogel

**Figure 10:**
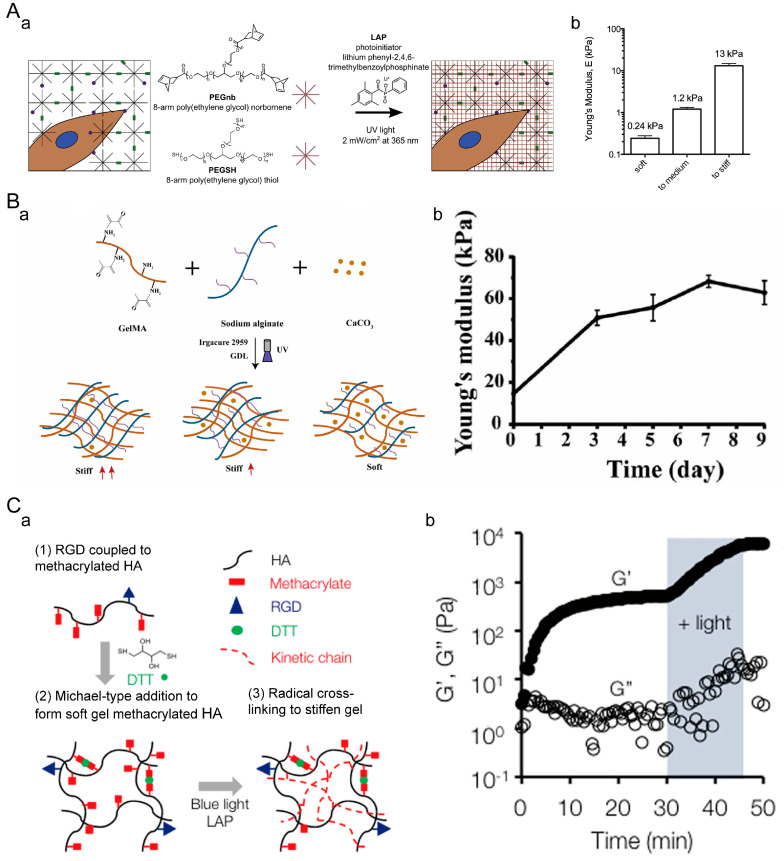
Dynamic stiffening hydrogel. (**A**) PEG hydrogel. (**a**) Principle of dynamic stiffening in PEG hydrogel. (**b**) Stiffness of hydrogel after dynamic stiffening. Adapted with permission from [162]. Copyright 2015, Elsevier. (**B**) GelMA/SA hydrogel. (**a**) Principle of dynamic stiffening in GelMA/SA hydrogel. (**b**) Stiffness variation during continuous stiffening of the hydrogel. Adapted with permission from [163]. Copyright 2022, Elsevier. (**C**) MeHA hydrogel. (**a**) Principle of dynamic stiffening in MeHA hydrogel. (**b**) Variation in storage modulus and loss modulus during dynamic stiffening of the hydrogel. Adapted with permission from [164]. Copyright 2016, Steven R. Caliari.

Dynamic stiffening hydrogels can simulate the increase in stiffness observed during the growth and development of biological tissues. Typically, this can be achieved by combining multiple crosslinking methods. Kelly M. Mabry et al. first prepared PEG-based hydrogels through photochemical thiol-ene polymerization (Figure 10A) [162]. They then swelled the hydrogels in a solution containing equimolar amounts of 8-arm PEGnb, 8-arm PEG-thiol, and LAP. Finally, in situ photopolymerization was performed, allowing for dynamic stiffening to different degrees. Zhenyin Chen et al. developed a UV-crosslinked GelMA/SA hydrogel hybrid system for dynamic stiffening (Figure 10B) [163]. The hydrogel is formed initially through UV crosslinking. Enclosed within the GelMA/SA hydrogel are CaCO_3_ and GDL, which enable the sustained release of calcium ions. The released calcium ions slowly crosslink with SA, resulting in a gradual and sustained increase in the stiffness of the matrix. Steven R. Caliari et al. used dithiothreitol (DTT) to crosslink methacrylated hyaluronic acid (MeHA) through a Michael addition reaction, resulting in a soft hydrogel (Figure 10C) [164]. Subsequently, the remaining methacrylate groups were further crosslinked through blue light irradiation, enabling dynamic stiffening.

#### 4.2.3. Hydrogel with Dynamic Reversible Stiffness

**Figure 11:**
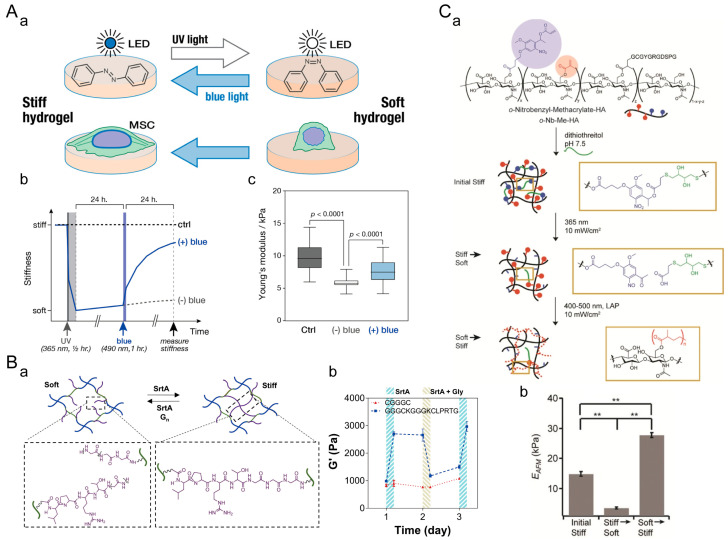
Hydrogels with dynamic reversible stiffness. (**A**) Polyacrylamide hydrogel. (**a**) Principle of reversible stiffness modulation in polyacrylamide hydrogels. (**b**) Adjusted time points for reversible stiffness modulation in the hydrogel. (**c**) Changes in stiffness during the process of stiffness modulation. Adapted with permission from [165]. Copyright 2018, American Chemical Society. (**B**) PEG-peptide hydrogel. (**a**) Principle of reversible stiffness modulation in PEG-peptide hydrogels. (**b**) Changes in stiffness during the process of stiffness modulation. Adapted with permission from [166]. Copyright 2019, Elsevier. (**C**) HA hydrogel. (**a**) Principle of reversible stiffness modulation of HA hydrogels. (**b**) Stiffness changes during the process of stiffness modulation. **: *p* < 0.01. Adapted with permission from [167]. Copyright 2017, Wiley-VCH GmbH.

Hydrogels with dynamic reversible stiffness can achieve cyclic control of stiffness through chemically reversible changes in the bonds. I-Ning Lee et al. reported a reversible hydrogel based on PA (Figure 11A) [165]. The gel contains a diazobenzene crosslinker, which softens the gel upon UV light irradiation and stiffens it upon visible light irradiation. Matthew R Arkenberg et al. designed a linear peptide with two SrtA substrates (GGG-CGGGC-LPRTG) (Figure 11B) [166]. The peptide was crosslinked into a hydrogel using thiol-ene photopolymerization. The N-terminal GGG and C-terminal LPRTG sequences serve as dangling chain motifs, allowing for SrtA-mediated secondary crosslinking and degradation, thus enabling the reversible stiffening and softening of the hydrogel. Adrianne M. Rosales and colleagues used ortho-nitrobenzyl acrylate and MeHA (Figure 11C) [167]. Dithiothreitol (DTT) preferentially crosslinks with the photodegradable ortho-nitrobenzyl group. These initial crosslinks can be degraded by controlling exposure to 365 nm light, and methacrylate can polymerize to stiffen the hydrogel at a desired time point. Compared to polymer hydrogels, designing protein-based dynamic hydrogels with tunable mechanical properties poses greater challenges. Protein hydrogels have the ability to finely tune their macroscopic mechanical characteristics in a rational manner [168]. Na Kong et al. constructed an engineered elastic protein, GB1-R-(GB1-GL5CC-I27-R)2, and employed a Ru^2+^-mediated photocrosslinking strategy to fabricate protein-based hydrogels [169]. These hydrogels can convert molecular-level folding–unfolding conformational changes into macroscopically reversible and adjustable mechanical properties.

## 5. The Influence of Dynamic Mechanical Microenvironments on Cell Behavior and Fate

Various hydrogel-based dynamic mechanical microenvironments have been developed to study the interactions between dynamic substrates and cells. Increasing evidence suggests that the dynamic mechanical properties of the matrix have a significant impact on cell behavior and fate (cell spreading, proliferation, migration, and differentiation) in both 2D and 3D cell cultures. The viscoelasticity and dynamic stiffness of the cellular microenvironment not only contribute to further exploration of the dynamic mechanical basis of the ECM but also aid in the development of tissue engineering. In this section, we will provide a detailed overview of cell responses to viscoelastic and dynamic stiffness microenvironments in both 2D and 3D cultures.

### 5.1. Viscoelastic Microenvironment Affects Cell Behavior and Fate

#### 5.1.1. Cell Spreading

**Figure 12:**
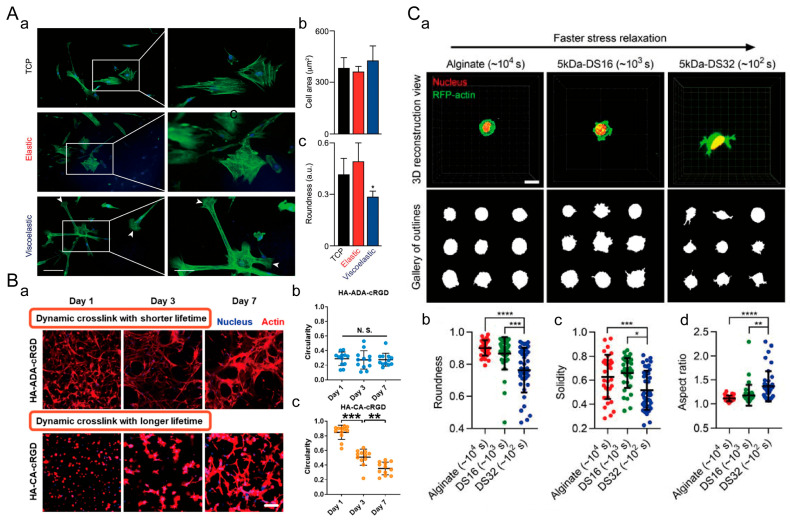
Regulation of cell spreading by ECM viscoelasticity. (**A**) Viscoelastic silk fibroin hydrogel for 2D cell culture. (**a**) Nuclear/cytoskeleton staining (scale bar: 20 μm). The white arrows indicate signs of membrane protrusion. (**b**) Cell area. (**c**) Cell roundness. *: *p* ≤ 0.05. Adapted with permission from [170]. Copyright 2021, American Chemical Society. (**B**) Viscoelastic hyaluronic acid (HA) hydrogel for 3D cell culture. (**a**) Nuclear/cytoskeleton (scale bar: 100 μm). (**b**) Cell roundness in dynamically crosslinked hydrogel with shorter lifetime. (**c**) Cell roundness in dynamically crosslinked hydrogel with longer lifetime. **: *p* < 0.01, ***: *p* < 0.001. N.S.: no statistical difference. Adapted with permission from [139]. Copyright 2021, Boguang Yang. (**C**) Viscoelastic sodium alginate hydrogel for 3D cell culture. (**a**) Morphology of 3T3 fibroblasts in sodium alginate hydrogels with different stress relaxation rates (scale bar: 10 μm). (**b**) Cell roundness. (**c**) Cell solidity. (**d**) Cell aspect ratio. *: *p* < 0.05, **: *p* < 0.01, ***: *p* < 0.001, ****: *p* < 0.0001. Adapted with permission from [171]. Copyright 2019, Elsevier.

Suttinee Phuagkhaopong et al. prepared a viscoelastic silk hydrogel by inducing hydrogen bond formation between crystalline regions of silk fibroin protein using ultrasound energy [170]. They investigated the influence of viscoelasticity on cell spreading on a 2D cell culture platform (Figure 12A). Compared to elastic hydrogels with the same stiffness, MSCs cultured on viscoelastic hydrogels exhibited a more elongated morphology and strong localized actin polymerization features on the third day. Cells cultured on viscoelastic hydrogels had larger cell areas and smaller roundness. This indicates that viscoelasticity promotes cell spreading in 2D culture. In 3D culture, Boguang Yang et al. prepared viscoelastic hyaluronic acid hydrogels with different loss moduli but the same storage modulus through host–guest crosslinking (Figure 12B) [139]. The spreading speed of MSCs in dynamic hydrogels with faster network association and dissociation rates is higher. Sungmin Nam et al. designed sodium alginate hydrogels with the same stiffness but different stress relaxation rates (Figure 12C) [171]. As the stress relaxation rate of ECM increases, 3T3 fibroblasts exhibit smaller roundness, smaller solid volume, and larger aspect ratios. Therefore, the viscoelasticity of the extracellular matrix promotes cell spreading in both 2D and 3D cell cultures.

#### 5.1.2. Cell Proliferation

**Figure 13:**
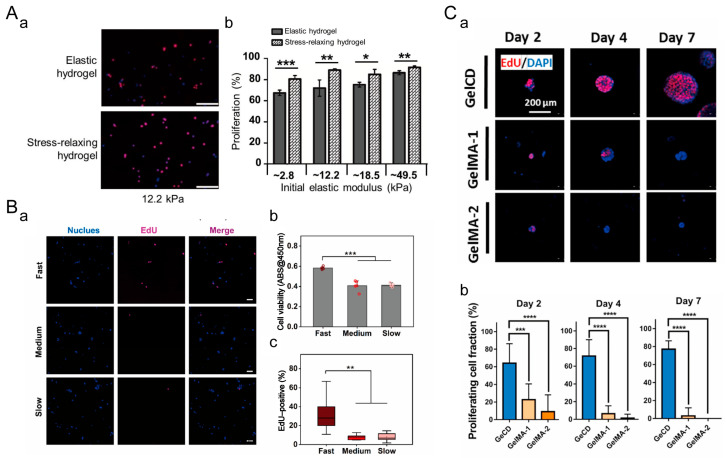
Regulation of cell proliferation by extracellular matrix viscoelasticity. (**A**) Sodium alginate hydrogel for 2D cell culture. (**a**) EdU staining image of C2C12 cells. EdU staining serves as an indicator of cell proliferation. EdU-positive cell nuclei are shown in red, while non-EdU-positive cell nuclei are shown in blue. Scale bar: 200 μm. *: *p* < 0.05, **: *p* < 0.01, ***: *p* < 0.001. Adapted with permission from [109]. Copyright 2017, Elsevier. (**b**) Quantification of C2C12 cell proliferation. (**B**) Viscoelastic sodium alginate hydrogel for 3D cell culture. (**a**) EdU staining image of MSCs. Scale bar: 20 μm. (**b**) Measurement of cell proliferation capacity using CCK-8 assay. (**c**) Percentage of EdU-positive MSCs in the hydrogel. **: *p* < 0.01, ***: *p* < 0.001. Adapted with permission from [172]. Copyright 2021, Elsevier. (**C**) Viscoelastic GelCD hydrogel for 3D cell culture. (**a**) EdU staining of mESCs. Scale bar: 200 μm. (**b**) Percentage of EdU-positive cell nuclei in the cell population. ***: *p* < 0.001, ****: *p* < 0.0001. Adapted with permission from [173]. Copyright 2022, Elsevier.

In a 2D cell culture, Aline Bauer et al. designed sodium alginate hydrogels with the same stiffness but different stress relaxation rates (Figure 13A) [109]. The proliferation rate of C2C12 mouse myoblasts on viscoelastic matrices was significantly higher than that on elastic matrices. Chuanchuan Lin et al. found that rapid stress relaxation upregulates the proliferation activity of MSCs in 3D, as observed through cck-8 and Edu staining (Figure 13B) [172]. Xiayi Xu et al. prepared a viscoelastic hydrogel (GelCD) through the supramolecular interaction between cyclodextrin and aromatic residues of gelatin [173]. Compared to elastic GelMA hydrogels, mESCs in GelCD hydrogels exhibited higher cell proliferation rates (Figure 13C). These findings indicate that the viscoelasticity of the ECM promotes cell proliferation in both 2D and 3D cell cultures.

#### 5.1.3. Cell Migration

**Figure 14:**
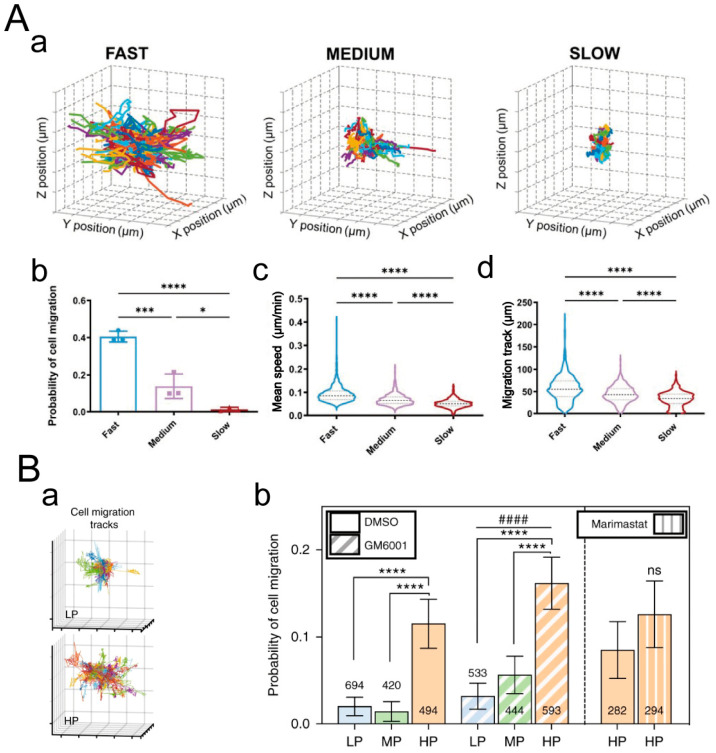
Regulation of cell migration by ECM viscoelasticity. (**A**) Viscoelastic PEG hydrogel for 3D cell culture. (**a**) Three-dimensional trajectory reconstruction of cell migration within the hydrogel. Grid size: 10 μm. 80 randomly selected cell migration track trajectories are shown for each condition. (**b**) Probability of cell migration. (**c**) Average migration velocity of cells and (**d**) length of cell migration trajectories. *: *p* < 0.05, ***: *p* < 0.001, ****: *p* < 0.0001. Adapted with permission from [175]. Copyright 2022, Wiley-VCH GmbH. (**B**) IPN hydrogels of reconstituted basement membrane (rBM) and alginate for 3D cell culture. (**a**) Reconstruction of 114D cell migration trajectories in the IPN hydrogel. Grid size: 10 μm. The migration of each cell is represented by trajectories in different colors. (**b**) Movement probability of cells in LP, MP, and HP IPN hydrogel with vehicle alone (DMSO) or protease inhibitors (10 μM GM6001 or 100 μM marimastat) added to the culture medium. ****: *p* < 0.0001, ^####^ *p* < 0.0001, ns: No statistical difference. Adapted with permission from [176]. Copyright 2018, Ovijit Chaudhuri.

In a 2D cell culture, Kolade Adebowale et al. designed sodium alginate hydrogel matrices with different stress relaxation rates [174]. On matrices with rapid stress relaxation, HT-1080 cells exhibited the highest average mean square displacement (MSD) and fastest migration velocity. In a 3D cell culture, Sauradeep Sinha et al. modified PEG and prepared dynamic hydrazone-crosslinked hydrogels using alkyl-hydrazone (AH) or benzyl-hydrazone (BH) linkages with different stress relaxation properties (Figure 14A) [175]. Compared to hydrogels with medium and slow stress relaxation rates, cells in hydrogels with fast relaxation properties exhibited enhanced migratory behavior, higher migration velocity, and longer migration trajectories. A study by Katrina M. Wisdom et al. showed that the migration probability of 114D cells in viscoelastic HP interpenetrating network (IPN) hydrogels with higher plasticity was approximately five times higher than that of cells in low plasticity LP IPN hydrogels (Figure 14B) [176]. These findings indicate that the viscoelasticity of ECM promotes cell migration in both 2D and 3D cell cultures.

#### 5.1.4. Cell Differentiation

**Figure 15:**
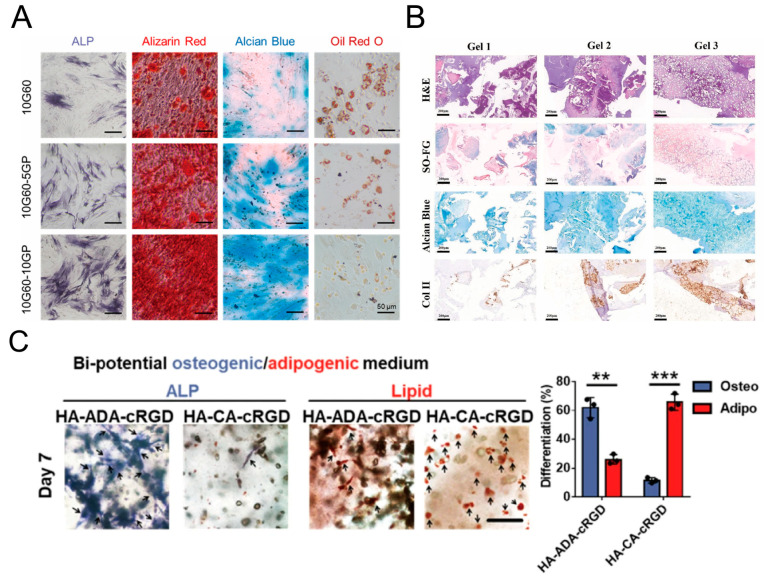
Regulation of stem cell differentiation by ECM viscoelasticity. (**A**) Viscoelastic hydrogel for 2D cell culture. Staining images of hydrogels using ALP, alizarin red, alcian blue, and Oil Red O. Scale bar: 50 μm. Adapted with permission from [177]. Copyright 2022, Changjiang Liu. (**B**) Supramolecular dual network (DN) hydrogel for 3D cell culture. Histological images of cartilage matrix secretion by MSCs encapsulated in DN hydrogels. Scale bar: 200 μm. (H&E = hematoxylin and eosin staining; SO-FG = safranin O–fast green staining). Adapted with permission from [178]. Copyright 2023, Elsevier. (**C**) HA hydrogel for 3D cell culture. Representative images of ALP and lipid staining and quantification of the percentage of hMSCs staining positive for osteogenic and adipogenic differentiation in HA-ADA-cRGD and HA-CA-cRGD hydrogels. **: *p* < 0.01, ***: *p* < 0.001. Adapted with permission from [139]. Copyright 2021, Boguang Yang.

In a 2D cell culture, Changjiang Liu et al. designed viscoelastic methacrylated gelatin (GelMA) hydrogels with independent and controllable stiffness and loss modulus (Figure 15A) [177]. Compared to the elastic hydrogel (10G60), the alkaline phosphatase (ALP), alizarin red, and alcian blue staining were more pronounced in the viscoelastic hydrogels (10G60-5 GP and 10G60-10 GP), while Oil Red O staining was weaker. As the loss modulus of the hydrogel increases, the osteogenic and chondrogenic differentiation abilities of BMSCs are enhanced, while the adipogenic differentiation tendency diminishes. In a 3D cell culture, Rong Yang et al. adjusted the ratio of host–guest crosslinked networks to covalently crosslinked networks to tailor the viscoelasticity of supramolecular DN hydrogels on demand. (Figure 15B) [178]. The staining results indicate that compared to the covalently crosslinked elastic hydrogel (Gel 1), the viscoelastic hydrogels (Gel 2 and Gel 3) significantly promote the deposition of sulfated GAG (a cartilage ECM marker) and secretion of type II collagen (Col-II). This suggests that the viscoelastic microenvironment is favorable for the chondrogenic differentiation of MSCs. Boguang Yang et al. found that hydrogels with a long crosslink lifetime (HA-CA-cRGD) were more effective in promoting MSC adipogenic differentiation. Hydrogels with a short crosslink lifetime (HA-ADA-cRGD) were more effective in promoting MSC osteogenic differentiation (Figure 15C) [139]. These findings indicate a consistent influence of viscoelasticity on stem cell differentiation in both 2D and 3D cell cultures. There is a mutual inhibition relationship between the signaling pathways involved in the adipogenesis and osteogenesis of stem cells. The adipogenic pathway can interfere with osteogenic signals and vice versa [179]. The viscoelastic dissipation of hydrogels can regulate intracellular integrin adhesion and actomyosin assembly, facilitating cell spreading and increasing cellular cytoskeletal tension. This, in turn, promotes nuclear translocation of the mechanosensitive transcriptional regulators YAP/TAZ, thereby enhancing the expression of genes related to stem cell chondrogenic and osteogenic differentiation [136,177,180].

### 5.2. The Dynamic Stiffness Affects Cell Behavior and Fate

#### 5.2.1. Cell Spreading

**Figure 16:**
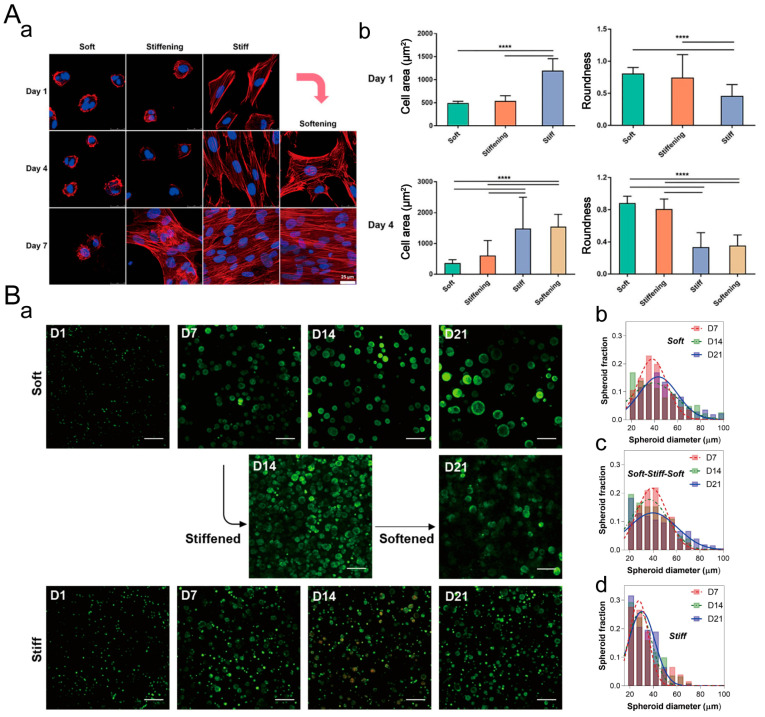
Dynamic stiffness of ECM regulates cell spreading. (**A**) Alginate hydrogels with dynamic stiffness used for 2D cell culture. (**a**) F-actin/nuclear staining images of hMSCs seeded on various hydrogels with different stiffnesses on day 1, 4, and 7. Scale: 25 μm. (**b**) Cell area and circularity on the hydrogels. ****: *p* < 0.0001. Adapted with permission from [181]. Copyright 2020, American Chemical Society. (**B**) Elastic PEG-peptide hydrogels with dynamic stiffness used for 3D cell culture. (**a**) Confocal images of COLO-357 cells encapsulated in the hydrogel. Scale: 200 μm. Diameter histograms of spheroids in soft (**b**), stiff (**c**), and reversibly stiffened and softened hydrogels (**d**). Adapted with permission from [166]. Copyright 2019, Elsevier.

In a 2D cell culture, a matrix with different stiffnesses leads to significant differences in cell spreading. Sustained stiffening or softening of sodium alginate hydrogels can be achieved by adding trace amounts of CaCl_2_ or sodium citrate to the culture medium [181]. They found that cell spreading was slower on soft matrices, while cells could spread quickly on stiffer matrices (Figure 16A). Due to the influence of mechanical memory, cells on softening matrices could still maintain a larger spreading area. Cells on stiffening matrices only started to spread rapidly after the fourth day, and it was not until the seventh day that they exhibited a fully spread cell morphology. The influence of stiffness on cell spreading in 3D cell culture is significantly different from that in 2D cell culture. Chun-Yi Chang et al. studied the effect of dynamic stiffness on cell spheroid volume (Figure 16B) [166]. When COLO-357 cell spheroids were cultured in soft hydrogels, the spheroid size increased steadily from 40 μm to 51 μm. Cell growth in stiff hydrogels was significantly impeded. The growth of cell spheroids was inhibited after stiffening (from D7 to D14), but subsequent softening of the hydrogel allowed the cell spheroids to grow again (from D14 to D21). Therefore, the influence of the dynamic stiffness of the extracellular matrix on cell spreading is diametrically opposed in 2D and 3D cell cultures.

#### 5.2.2. Cell Proliferation

**Figure 17:**
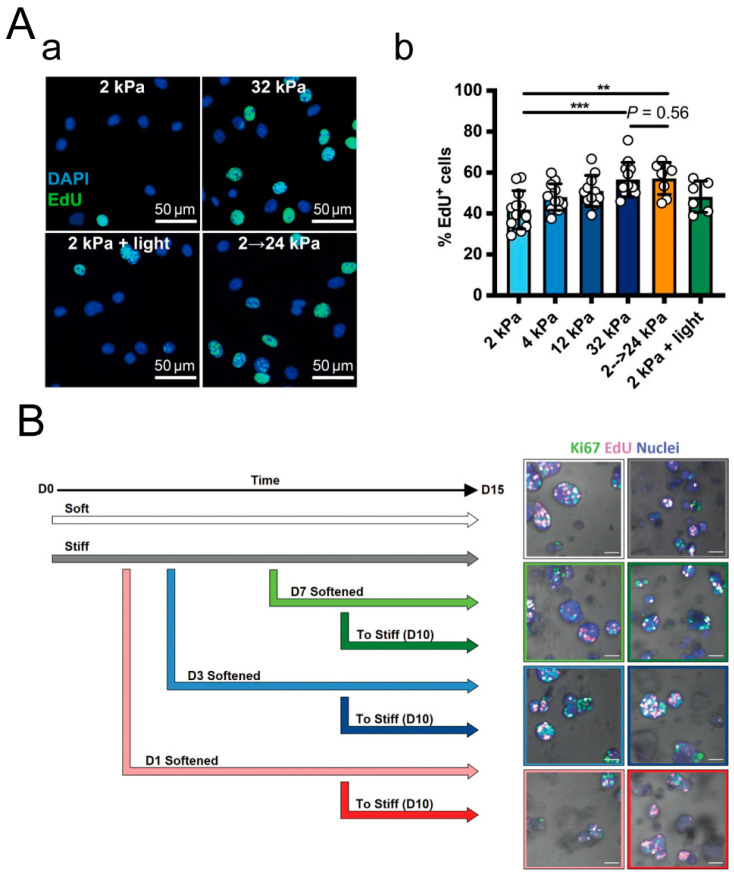
Dynamic stiffness of ECM regulates cell proliferation. (**A**) PEG hydrogel with dynamic stiffness used for 2D cell culture. (**a**) EDU staining of C2C12 cells representing cell proliferation. (**b**) Proportion of EdU-positive cells on the hydrogel. **: *p* < 0.01, ***: *p* < 0.001. Adapted with permission from [104]. Copyright 2021, Jason S. Silver. (**B**) Sequential modulation of (PEG)-peptide hydrogel during 3D cell culture: experimental setup and outputs, as well as Ki67 and EdU staining (representing cell proliferation) of T47D cells. Scale bar: 50 μm. Adapted with permission from [160]. Copyright 2022, Wiley-VCH GmbH.

In a 2D cell culture, Jason S. Silver et al. developed a hydrogel formed through a copper-free click reaction between azide groups and activated cyclooctyne (DBCO), known as SPAAC (Strain-promoted Azide-Alkyne Cycloaddition). This hydrogel can undergo secondary photopolymerization of unreacted alkynes for in situ stiffening, mimicking ECM stiffening [104]. They found that C2C12 cell proliferation rates were higher on a stiffer substrate (Figure 17A). There was no significant difference in the cell proliferation rate between dynamically stiffened (increased from 2 kPa to 24 kPa) and statically stiff (32 kPa) substrates. This indicates that the dynamic stiffening of the substrate enhances cell proliferation. In a 3D cell culture, Katherine L et al. found that the softening of the hydrogel promotes the proliferation of T47D cells compared to statically stiff hydrogels (Figure 17B) [160]. Subsequent stiffening, on the other hand, leads to reduced cell proliferation. This may be due to the density of the matrix restricting cell growth. Therefore, the dynamic stiffness of the ECM has completely opposite effects on cell proliferation in 2D and 3D cell cultures.

#### 5.2.3. Cell Differentiation

**Figure 18:**
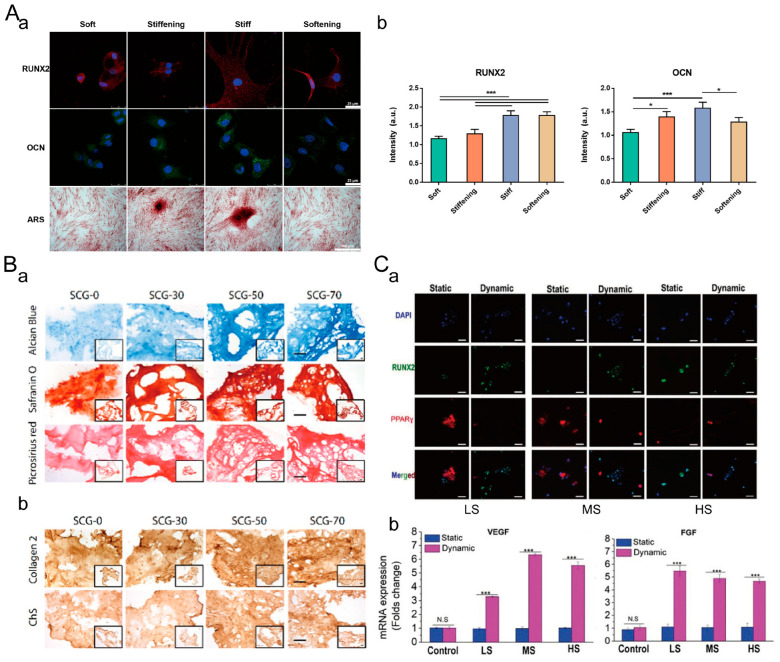
ECM with dynamic stiffness regulates stem cell differentiation. (**A**) Dynamic stiffness alginate hydrogel used for two-dimensional cell culture. (**a**) Immunofluorescence staining images of hMSCs on various hydrogels after 5 and 10 days, showing RUNX2 (red), OCN (green), and cell nucleus (blue), indicating early-stage osteogenic differentiation and late-stage osteogenic differentiation. Scale bar: 25 μm. Representative images of hMSCs stained with ARS after 14 days, demonstrating calcium deposition. Scale bar: 500 μm. (**b**) Quantitative fluorescence intensity of RUNX2 and OCN. *: *p* < 0.05, ***: *p* < 0.001. Adapted with permission from [181]. Copyright 2020, American Chemical Society. (**B**) Silk fibroin/carboxymethyl cellulose/gelatin (SCG) hydrogel with dynamic stiffness used for three-dimensional cell culture. (**a**) Images of alcian blue, safranin O, and picrosirius red staining of SCG hydrogel sections. (**b**) Immunohistochemical staining images of collagen II and chondroitin sulfate of SCG hydrogel sections. Scale bar: 100 μm; insets: 200 μm. Adapted with permission from [183]. Copyright 2022, American Chemical Society. (**C**) GelMA hydrogel with dynamic stiffness used for three-dimensional cell culture. (**a**) Immunofluorescent images of osteogenic biomarker RUNX2 (green) and adipogenic biomarker PPARγ (red) in LS, MS, and HS hydrogels under static and dynamic culture conditions. (**b**) Gene expression of angiogenic biomarkers VEGF and FGF. *** *p* < 0.001. N.S: no statistical difference. Adapted with permission from [184]. Copyright 2019, Wiley-VCH GmbH.

In previous studies, the stiffness of static hydrogels has been shown to influence the lineage specification of stem cells. For example, on a two-dimensional hydrogel substrate, a stiff matrix favors stem cell osteogenic differentiation, while a soft matrix promotes adipogenesis [182]. Mesenchymal stem cells (MSCs) cultured on soft (0.1–1 kPa), medium (8–17 kPa), and stiff (25–40 kPa) polyacrylamide (PAA) gels mimicking brain, skeletal muscle, and pre-mineralized bone environments, respectively, differentiate into neuronal cells, osteoblasts, and skeletal muscle cells [62]. Current research suggests that the dynamic stiffness of hydrogels also affects stem cell differentiation. In a 2D cell culture, Dan Wei et al. investigated the influence of the dynamic stiffness of the matrix on hMSCs osteogenic differentiation (Figure 18A) [181]. After 5 days, there was no significant difference in RUNX2 expression between the MSC cultured on stiff hydrogels and softening hydrogels, both of which were higher than that in soft hydrogels and stiffening hydrogels. After 10 and 14 days, MSCs cultured on stiffening hydrogels exhibited significantly enhanced OCN expression and calcium deposition but still lower than those of stiff hydrogels. This suggests that the early low stiffness in the stiffening system delayed hMSCs osteogenic differentiation. OCN expression remained high in the softened hydrogels, indicating that early high stiffness in the softening system retained the effect on osteogenic differentiation. Therefore, the osteogenic differentiation of hMSCs is not solely determined by the real-time stiffness of the matrix but also influenced by past mechanical contacts. In a 3D cell culture, Aman Mahajan et al. demonstrated that compared to the static hydrogel (SCG-0), dynamic stiffening hydrogels (SCG-30, SCG-50, SCG-70) significantly enhanced the deposition of sGAG (alcian blue and safranin O staining images) and collagen (picrosirius red staining images) (Figure 18B) [183]. The deposition of type II collagen (Col-II) and chondroitin sulfate in SCG-50 and SCG-70 hydrogels was also significantly increased compared to SCG-0 hydrogel. Overall, the dynamic stiffening and contraction of the hydrogels promote the chondrogenic differentiation of infrapatellar-fat-pad-derived mesenchymal stem cells (IFP-MSCs). Jianguang Zhang et al. embedded nanogels of stimuli-responsive poly (N-isopropylacrylamide-co-2-hydroxyethyl methacrylate) (P-NIPAM-HEMA) into GelMA hydrogels, allowing for reversible hydrogel stiffness modulation by changing the environmental temperature. They developed low stiffness (LS), medium stiffness (MS), and high stiffness (HS) hydrogels with a temperature-responsive storage modulus (G′) that could reversibly change (Figure 18C) [184]. Immunofluorescent staining of osteogenic biomarker RUNX2 and adipogenic biomarker PPARγ indicated that under static conditions (with a constant temperature of 37 °C), hMSCs in the soft hydrogel tended to undergo adipogenic differentiation, while under dynamic culture conditions (with temperature cycling from 25 °C to 37 °C), hMSCs tended to undergo osteogenic differentiation. Dynamic mechanical stimulation can activate YAP by enhancing actomyosin activity, and the degree of YAP activation is positively correlated with the number of mechanical stimulation cycles. This promotes the osteogenic differentiation of stem cells. The gene expression of vascular endothelial growth factor (VEGF) and fibroblast growth factor (FGF) also suggests that dynamically tuned hydrogels are effective strategies for guiding hMSC sphere fate and enhancing vascularization potential. Therefore, the dynamic stiffness of the ECM can effectively modulate stem cell differentiation in both 2D and 3D cell cultures.

## 6. Conclusions and Future Perspectives

In the past few decades, research on controlling the mechanical properties of biomaterials has rapidly advanced, facilitating our understanding of the interactions between the cells and the matrix. Dynamic mechanical properties are a universal characteristic of biological tissues and organs, and cells sense and respond to their dynamic mechanical microenvironment to achieve normal cellular functions. Therefore, research in this field has gradually shifted from studying static biomaterials to materials with dynamic mechanical properties. The development of dynamic hydrogels (viscoelasticity and dynamic stiffness) to mimic native 3D cellular microenvironments and study their regulation of cellular behavior is of significant importance. The crosslinked networks of these hydrogels allow control over the interaction between cells and the surrounding environment, and increasing evidence suggests that cells respond to time-dependent dynamic mechanical cues. In fact, recent findings have demonstrated that viscoelasticity and dynamic stiffness can significantly alter cell behavior and regulate their fate in both 2D and 3D cell cultures. For instance, dynamic stiffening and stress relaxation can promote the osteogenic differentiation of MSCs [180,185]. These findings provide new strategies for the diagnosis and treatment of clinical diseases in the future.

The further development of dynamic mechanical hydrogels faces both challenges and opportunities. Recently, several studies have attempted to uncover the pathways by which cells respond to the dynamic mechanical microenvironment. However, most of these pathways are focused on mechanosensitive receptors based on integrins, actin cytoskeletal rearrangement, and mechanical signaling through nuclear localization of YAP/TAZ [139,149,186]. Indeed, the process of cells sensing mechanical cues and translating them into chemical signals is undoubtedly more complex. By further investigating how mechanical stimuli affect cell behavior, researchers will be able to advance the design of cell-based biomedical therapies in the future.

Furthermore, current research on the dynamic mechanical properties of the ECM mostly focuses on two aspects: viscoelasticity and dynamic stiffness. However, the viscoelasticity of biological tissues may gradually change during development or disease processes, thereby affecting cell behavior and fate. For example, the viscosity of rabbit bones decreases by 20% during the first 6 months of their life [100]. The viscosity (G″/G′) of the mouse brain also gradually decreases within 24 h after death [187]. In chronic pulmonary arterial hypertension, the viscosity of the main pulmonary artery significantly decreases compared to the healthy state [188]. Therefore, studying the dynamic changes in the viscoelasticity of biomimetic tissues and their impact on cell behavior can provide a theoretical foundation for the design of tissue engineering scaffold materials and has important research and clinical applications. Only a few studies so far have focused on the dynamic modulation of ECM viscoelasticity. For example, Ryota Tamate et al. developed a cell-compatible and dynamically adjustable viscoelastic hydrogel [189]. It is composed of an ABA triblock copolymer and can significantly alter the viscoelasticity of the hydrogel simply by UV irradiation. Lu Pang et al. prepared a magnetic hydrogel scaffold encapsulating Fe_3_O_4_ nanoparticles [190]. The viscoelasticity of the hydrogel can be changed by altering the applied external magnetic field strength. However, although these systems can change the viscoelasticity of the hydrogel, they also simultaneously alter the stiffness of the hydrogel. This introduces the confounding factor of stiffness changes when studying the effects of dynamic viscoelasticity on cell behavior and fate. Therefore, developing a hydrogel system with invariant stiffness and dynamically adjustable viscoelasticity would eliminate the influence of stiffness changes on cellular activities and accurately explore the effects of dynamic viscoelasticity on cell behavior and fate.
